# Stereolithography (STL) measurement rubric for the evaluation of craniomaxillofacial STLs

**DOI:** 10.1186/s41205-022-00151-x

**Published:** 2022-08-08

**Authors:** Henra Muller, Annabel Fossey

**Affiliations:** 1 Department of Clinical Imaging Sciences, School of Clinical Medicine, Faculty of Health Sciences, University of the Free State, Bloemfontein, South Africa; 2 Department of Clinical Sciences, Faculty of Health and Environmental Sciences, Central University of Technology Free State, C/o Park Road & President Brand Street, Bloemfontein, 9300 South Africa; 3 Graduate Mastery, Randburg, South Africa

**Keywords:** CT image quality, Internal cranial prosthesis, STL, STL measurement rubric, 3D printing

## Abstract

**Background:**

Facial deformities often demand reconstructive surgery and the placement of three-dimensional (3D) printed craniomaxillofacial prostheses. Prostheses manufacturing requires patients’ computed tomography (CT) images. Poor quality images result in incorrectly sized prostheses, necessitating repeat imaging and refitting. The Centre for Rapid Prototyping and Manufacturing (CRPM) produces most facial prostheses in South Africa but does not have a prescribed optimised CT protocol. Therefore, this study was undertaken.

**Methods:**

A collection of CRPM STLs used in the design and manufacturing of craniomaxillofacial prostheses is available. The image quality of stereolithography (STL) files of CRPM CT scans was evaluated to determine what constitutes good image quality. This collection was scrutinised for inclusion in the image quality evaluation. After scrutiny, 35 STLs of individuals ≥15 years of age were selected and included metadata attached to the DICOM file. Furthermore, only STLs created without manipulation by the same designer were included in the collection. Before the qualitative evaluation of the STLs, eight different critical anatomical reference points (CARPs) were identified with the assistance of an expert team. A visual acuity rating scale of three categories was devised for each CARP, where 1 was allocated to poor visual acuity, 2 to partial, and 3 to good visual acuity. Similarly, rating scales were devised for the presence of concentric rings and the overall impression score awarded by the two designers involved in the design and manufacturing of the prostheses. This stereolithography measurement rubric (SMR) was then applied to the 35 STLs by a team of three experts, including the two designers, during a structured evaluation session. The scores were used to calculate summary and inferential statistics.

**Results:**

Scores grouped around the central rating of partial visual acuity. The three evaluators’ mean total CARP scores ranged from 13.1 to 14.4 (maximum possible score 24), while the mean total CARP + ring scores ranged from 15.8 to 17.1 (maximum possible score 27). No significant differences were detected between the evaluators’ scores.

**Conclusion:**

This SMR appears to be the first of its kind. This image quality assessment of STLs provides the groundwork for finer CT image quality evaluation to formulate a CT imaging protocol for the CRPM to design and manufacture accurate internal cranial prostheses.

## Background

Facial deformities and disfigurements may have a profound psychosocial impact on an individual. The visibility of disfigurement and being perceived as ‘abnormal’ by society can present various challenges. People with disfigurements often experience rejection by society, who treats them as outcasts, resulting in their suffering from anxiety, severe depression and poor self-esteem [1, 2]. The cause of facial disfigurements can be either congenital or acquired. Most facial disfigurements are acquired, while malformed or the absence of facial features is examples of congenital disfigurements. Acquired disfigurements are mainly the result of systemic pathologies, for example, cancer, but could result from traumatic events, including motor vehicle accidents and assaults [3]. In South Africa, statistics show that facial trauma injuries mainly result from the high prevalence of road traffic accidents, assaults and shack fires. Shack fires and primus stoves are the leading causes of burn injuries in South Africa, which can also cause facial disfigurements [4].

Patients with facial disfigurements often seek medical interventions to improve their physical appearance. While some restorative interventions can be performed for improved functional purposes, such as chewing, most interventions are for aesthetic reasons [5]. Improvement of such deformities may require cranial reconstructive surgery and placement of implants or prostheses. Maxillofacial prostheses are considered by many the primary choice of treatment for functional rehabilitation, aesthetic reconstruction and rebuilding a patient’s confidence, and can either be external, internal or both [6]. The process of manufacturing a maxillofacial prosthesis involves the creation of a three dimensional (3D) solid object from a 3D digital file through the process of additive manufacturing (AM) [7]. A good and appropriate prosthesis results in patients demonstrating improved mental health, social engagement and the ability to lead productive lives [8]. Manufacturing craniomaxillofacial prostheses require computed tomography (CT) images of the facial area, from which a prosthesis is designed for 3D printing.

The quality of preoperative CT images is crucial, as it is used to plan and print an implant unique to an individual. The accuracy of the 3D printed model of a patient’s anatomy has a major influence when selecting appropriate treatment options by clinicians. When suboptimal CT images are used for the reconstructive model design and manufacturing, it could result in incorrect sizing of the printed device, which could have detrimental effects during surgery and may require repeated imaging and refitting, which could cause patient distress [9]. Historically, the end goal for CT imaging was for the diagnosis of disease and not necessarily the design and manufacturing of a 3D printed implant. Manmadhachary [10] stated that the accuracy of a 3D medical model generated from CT images has not been investigated sufficiently yet.

The Centre for Rapid Prototyping and Manufacturing (CRPM) at the Central University of Technology, Free State (CUT) in Bloemfontein, South Africa, is responsible for most craniomaxillofacial prostheses design and manufacturing in South Africa. Currently, the CRPM does not have a prescribed optimised CT imaging protocol specifically for the design and manufacturing of internal cranial prostheses. The need for standardisation and optimisation in protocols remains, as CT scanners differ in their capabilities and various clinical indications require unique protocols [11]. The adoption of standard imaging protocols, especially in specialised modalities such as CT and magnetic resonance imaging (MRI), may reduce the chance of error or discrepancy in some areas of radiology practice [12]. To develop an optimised CT protocol, understanding what constitutes a good quality CT scan is thus required. Therefore, this study was undertaken to devise a measurement rubric that can be used to evaluate the image quality of STLs generated from CT scan Digital Imaging and Communications in Medicine (DICOM) files. This study formed part of a larger study with the end goal to produce an optimised CT protocol with CT parameter threshold values to design and manufacture craniomaxillofacial prostheses at CRPM. Towards this end goal, an STL collection was subjected to different image quality evaluations, of which the first was to apply a rubric to evaluate STL image quality.

## Methods

### Selection of STLs for image quality measurement

At the CRPM, access to a collection of STLs used to design and manufacture craniomaxillofacial prostheses was available. This collection comprised 48 STLs that were derived from original CT DICOM files, to which access could not be obtained. The collection of STLs was scrutinised for their appropriateness for the study by applying the following exclusion criteria:

(i) non-CT data images, such as MRI and cone-beam CTs;
(ii) duplicate STLs; and
(iii) STLs without CT scan metadata.

Once all the non-CT data images and duplicates were removed, the resultant STL data collection was scrutinised for age appropriateness and the presence of CT scan metadata. To ensure the most uniform collection of STLs for the study, only STLs of patients 15 years or older were included in the STL data collection (*n* = 35). STLs of patients younger than 15 were deemed inappropriate, as the CT parameter selection may differ greatly from that of the CT parameter selection for adult patients [13].

To further ensure uniformity, only STLs created without manipulation by the same designer were included in the STL collection. The designer opened the original CT DICOM files in Materialise Mimics® Medical version 24.0 and Materialise 3-matic® (Materialise NV; Leuven. Belgium) and segmented the data by applying the default threshold settings (a minimum of 226 Hounsfield unit [HU] value and a maximum of 3071 HU) with region growing. Artificial intelligence (AI) automated segmentation was not applied in the process. The ‘optimal’ STL quality setting was selected during the “calculate meshing” step. The computer hardware used to create the STLs met the minimum requirements stipulated by Mimics. When creating the STLs, no artifacts were removed by the designer. Because of the uniform treatment of the CT DICOM files during the creation of the STLs, this collection of STLs was deemed appropriate to test a measurement rubric that could be used to evaluate the image quality of STLs.

### Measurement of STL image quality

For the measurement of STL image quality, three steps were followed. In the first step, appropriate image quality variables were identified and thereafter referred to as evaluation items. In the second step, an STL measurement rubric (SMR) was formulated to measure the respective evaluation items of STL image quality. In the last step, the SMR was applied to measure the image quality of the selected STLs. An expert evaluation task team was constituted and included the two designers responsible for prostheses design at CRPM, a specialist who had extensive experience working with similar data sets. After a lengthy discussion, the expert evaluation task team agreed that five evaluation items should be used for STL image evaluation (Table 1). Two additional image quality evaluation items were added to the list to provide a more robust measurement of the respective STLs; one relating to the presence or absence of concentric rings on an STL, and the other relating to the overall impression of the two designers who used the STLs in prosthesis design.

**Table 1:** Evaluation items selected for STL image quality evaluation

Evaluation item	Reason for selecting evaluation item
Critical anatomical reference point (CARP)	For the evaluation of the image quality of an STL, specific cranial anatomical landmarks were necessary. The purpose of these anatomical landmarks was to make it possible to differentiate between different levels of STL image quality. For example, the different degrees of clarity of the delineation of the orbital foramina and the mandibular canal could be used as a means to evaluate STL image quality.
Ring artifact	For the evaluation of the image quality of an STL, the presence or absence of a ring artifact could be used as an evaluation item of image quality. The presence of these concentric rings could hamper the design process and be indicative of reduced STL image quality.
Overall impression of STL	For the evaluation of the image quality of an STL, the overall impression of the designers who used the STL for prosthesis design, could be indicative of STL image quality. Their experience on the ease of use of an STL in the design of the prosthesis could make a valuable contribution to the evaluation of STL image quality.
Composite evaluation item(s) that include all CARP measurements	For the evaluation of the image quality of an STL, a composite measurement (description) may be an advantage because the sum of all the CARP measurements could be a more comprehensive measurement (description) of STL image quality.
Composite evaluation item(s) that include all CARP measurements and presence or absence of ring artifact measurement	For the evaluation of image quality of an STL, a composite measurement (description), which includes the presence or absence of ring artifact, may be an advantage because the sum of all these measurements could be a more comprehensive measurement (description) of STL image quality.

Several CARPs were identified for the evaluation of the image quality of the STLs. In the event that some of the CARPs could be missing from a CT scan, the expert evaluation task team suggested that more than five CARPs should be identified to ensure that a sufficient number of measurements could be generated for each of the CT scans. Thus, the team suggested eight different CARPs of various anatomical regions of the cranium, including cranial foramina, cranial sutures and particular structures such as the mandible and the teeth (Table 2).

**Table 2:** Descriptions and pictures of the different CARPs used for STL image quality evaluation

CARP	Description of CARP	Image of CARP
1. Cranial sutures	The cranial sutures refer to a fibrous joint that holds bony plates together and only occurs in the cranium.	Gray (1918) [14]
2. Head of the mandible	The head of the mandible refers to the condyle, which presents an articular surface for articulation with the articular disk of the temporomandibular joint.	https://media.cheggcdn.com/media/76c/76c5717c-0c6b-40ad-bee9-4ad78e2bc1fb/phpoRCXA2.png Chegg® Study (2021)
3. Temporomandibular fossa separation	The temporomandibular fossa separation refers to the boundary between the temporomandibular fossa and the condylar head.	Gray (1918) [14]
4. Supraorbital foramina	The supraorbital foramina refer to the bilateral openings in the skull’s frontal bone located above the supraorbital margin of the orbits.	Gray (1918) [14]
5. Infraorbital foramina	The infraorbital foramina refer to the bilateral openings in the skull’s maxillary bone located below the infraorbital margin of the orbits.	Gray (1918) [14]
6. Mental foramina	The mental foramina refer to the two openings located on the mandible’s anterior surface.	Gray (1918) [14]
7. Teeth [15]^a^	The teeth refer to all the different types of teeth present in the mandible and maxilla.	https://pubs.rsna.org/cms/10.1148/rg.307105026/asset/images/medium/105026fig04a.jpeg
8. Mandibular canal	The mandibular canal refers to a canal within the mandible containing the inferior alveolar nerve, inferior alveolar artery and inferior alveolar vein.	https://media.cheggcdn.com/media/76c/76c5717c-0c6b-40ad-bee9-4ad78e2bc1fb/phpoRCXA2.png Chegg® Study (2021)

^a^Image used with permission from Saavedra-Abril JA, Balhen-Martin K, Zaragoza-Velasco K, Kimura-Yahama ET, Saavedra S, Stoopen ME. Dental multisection CT for the placement of oral implants: technique and applications. Radiographics. 2010;30(7):975–1991, page 1978. Copyright holder: Radiological Society of North America (RSNA) [15]

The SMR was created to measure the respective evaluation items in consultation with the evaluation team. The team members agreed that a 3-point rating scale that focused on the visual acuity of the respective CARPs would be appropriate for measuring the image quality of the STLs. The 3-point rating comprised a rating of “1” that indicated poor visual acuity; “2” that indicated partial visual acuity; and “3” that indicated good visual acuity of a particular CARP. Table 3 provides the SMR containing the rating scales and descriptions for the ten evaluation items used in the measurement of the image quality of the STLs. For the measurement of the STL image quality, three evaluators were identified and included the two designers and a specialist member of the expert evaluation task team. At a meeting, the evaluators were tasked to score each STL individually by applying the guidelines of the SMR. The scores were thereafter captured on Excel spreadsheets designed for the study.

**Table 3:** STL measurement rubric consisting of the rating scales and their descriptions for the image quality measurement of the STLs

Evaluation item	Qualitative measurement rating scale		
	Poor visual acuity Rating = 1	Partial visual acuity Rating = 2	Good visual acuity Rating = 3
1. Cranial sutures	Cranial sutures demonstrate poor visual acuity and appear to be smooth.	Cranial sutures demonstrate partial visual acuity and appear to have some definition but still unclear.	Cranial sutures demonstrate good visual acuity and appear clearly defined.
2. Head of the mandible	Head of the mandible demonstrates poor visual acuity and does not show clear delineation of the different individual anatomical structures.	Head of the mandible demonstrates partial visual acuity and shows some delineation of the different individual anatomical structures but not clearly.	Head of the mandible demonstrates good visual acuity shows clear delineation of the different individual anatomical structures.
3. Temporomandibular fossa separation	The boundary between the fossa and the adjacent skull demonstrates poor visual acuity. No clear delineation exists between the margins of the fossa and adjacent anatomical structures.	The boundary between the fossa and the adjacent skull demonstrates partial visual acuity. Incomplete separation exists between the margins of the fossa and adjacent anatomical structures.	The boundary between the fossa and the adjacent skull demonstrates good visual acuity. Complete and clear separation exists between the margins of the fossa and adjacent anatomical structures.
4. Supraorbital foramina	Supraorbital foramina demonstrate poor visual acuity and are not clearly delineated.	Supraorbital foramina demonstrate partial visual acuity and are partially delineated.	Supraorbital foramina demonstrate good visual acuity and are clearly delineated.
5. Infraorbital foramina	Infraorbital foramina demonstrate poor visual acuity and are not clearly delineated.	Infraorbital foramina demonstrate partial visual acuity and are partially delineated.	Infraorbital foramina demonstrate good visual acuity and are clearly delineated.
6. Mental foramina	Mental foramina demonstrate poor visual acuity and are not clearly delineated.	Mental foramina demonstrate partial visual acuity and are partially delineated.	Mental foramina demonstrate good visual acuity and are clearly delineated.
7. Teeth	Individual teeth demonstrate poor visual acuity. Poor discrimination between individual teeth.	Individual teeth demonstrate partial visual acuity. Partial discrimination between individual teeth.	Individual teeth demonstrate good visual acuity. Good discrimination between individual teeth.
8. Mandibular canal (inferior alveolar nerve)	The mandibular canal demonstrates poor visual acuity and is not clearly delineated.	The mandibular canal demonstrates partial visual acuity and is partially delineated.	The mandibular canal demonstrates good visual acuity and is clearly delineated.
9. Concentric rings visible (rings score)	Present = 0	Absent = 3	
10. Overall impression of the designer	STL poses relative difficulty in the design of the prosthesis.	STL poses some difficulty in the design of the prosthesis.	STL poses no difficulty in the design of the prosthesis.

### Statistical analysis

Several statistical analyses were performed on the measurements of the different evaluation items used to measure the image quality of the respective STLs. Summary statistics were calculated for all evaluation items. Inferential statistics were also performed on the measurements to ascertain to what extent the measurements of the three evaluators were consistent with one another. Hence the following hypotheses were derived:

- *H1:* If the differences in measurements by the three evaluators for the individual *CARPs* were 5% or less, then the differences were not because of random fluctuations. This hypothesis was tested through the application of the Kruskal-Wallis one-way analysis of variance (ANOVA).
- *H2:* If the differences in measurements by the three evaluators for the *Total CARP score* were 5% or less, then the differences were not because of random fluctuations. This hypothesis was tested through the application of the Kruskal-Wallis one-way ANOVA.
- *H3:* If the differences in measurements by the three evaluators for the *Total CARP + ring score* were 5% or less, then the differences were not because of random fluctuations. This hypothesis was tested through the application of the Kruskal-Wallis one-way ANOVA.
- *H4:* If the differences in measurements by the two designer evaluators for the *Overall impression score* were 5% or less, then the differences were not because of random fluctuations. This hypothesis was tested through the application of the Mann-Whitney U test.

To ascertain if an association existed between the *Overall impression score* of the design evaluators and the two evaluation items, *Total CARP score* and *Total CARP + ring score,* Spearman’s rank correlation coefficients were calculated. Thus, the following hypotheses were derived:

- *H5:* If the *Total CARP score* is associated with the *Overall impression score*, then a high *Total CARP score* will result in a high *Overall impression score*.
- *H6:* If the *Total CARP + ring score* is associated with the *Overall impression score*, then a high *Total CARP + ring score* will result in a high *Overall impression score*.

### Classification of STL image quality

For the classification of the image quality of the STLs, a systematic classification process was required so that the STLs could be classified into a number of image quality categories. It was therefore decided that three broad image quality categories would be appropriate for the evaluation of the STLs. For the STL image quality classification, the measurements of the evaluation items, *Total CARP score* and *Total CARP + ring score*, were deemed appropriate. Both these evaluation items encompass a more or less holistic evaluation of an STL’s image quality. The *Total CARP score* is a composite value of all the CARP measurements, while *Total CARP + ring score* is a composite value of all the CARP measurements and whether rings were present on an STL. Thus, a systematic step-by-step process was devised to guide the classification of the STL image quality. The systematic step-by-step process was as follows:

1. Firstly, the rating scores of the three evaluators for the evaluation items *Total CARP score* and *Total CARP + ring score* were listed for each STL;
2. The *Total CARP score* values were then used to classify the STLs into three broad image quality categories, low (L), medium (M) and high (H), where a rating value of 1–8 implied low STL image quality, 9–16 medium STL image quality and 17–24 high STL image quality;
3. The *Total CARP + ring score* values were also used to classify the STLs into three broad image quality categories, L, M and H, where a rating value of 1–9 implied low STL image quality, 10–18 medium STL image quality and 19–27 high STL image quality; and
4. To obtain the final image quality classification category for an STL, the classification categories were compared for each STL and the final image quality classification category awarded to an STL by choosing the highest image quality category. For example, a classification of H would be awarded to an STL when at least one of either the *Total CARP score* or *Total CARP + ring score* was categorised as H.

### Ethical considerations

The study was approved by the Health Sciences Research Ethics Committee (HSREC; reference number UFS-HSD2020/1719/2601) of the University of the Free State and the Free State Province Department of Health, South Africa. Furthermore, because of the retrospective nature of the study, patient informed consent was not required. All CT scan data from CRPM used during the research study were anonymised and no personal information of any of the patients was disclosed.

## Results and discussion

### STL image quality analysis

Through the application of the SMR, the expert evaluation team graded the different CARPs on the STLs in terms of visual acuity. By applying the 3-point rating scale of the SMR, the team was able to grade each of the CARPs in terms of visual acuity into categories indicating poor, partial and good visual acuity. To better understand the visual representation of these ratings, representative examples were selected and are illustrated in Table 4.

**Table 4:**
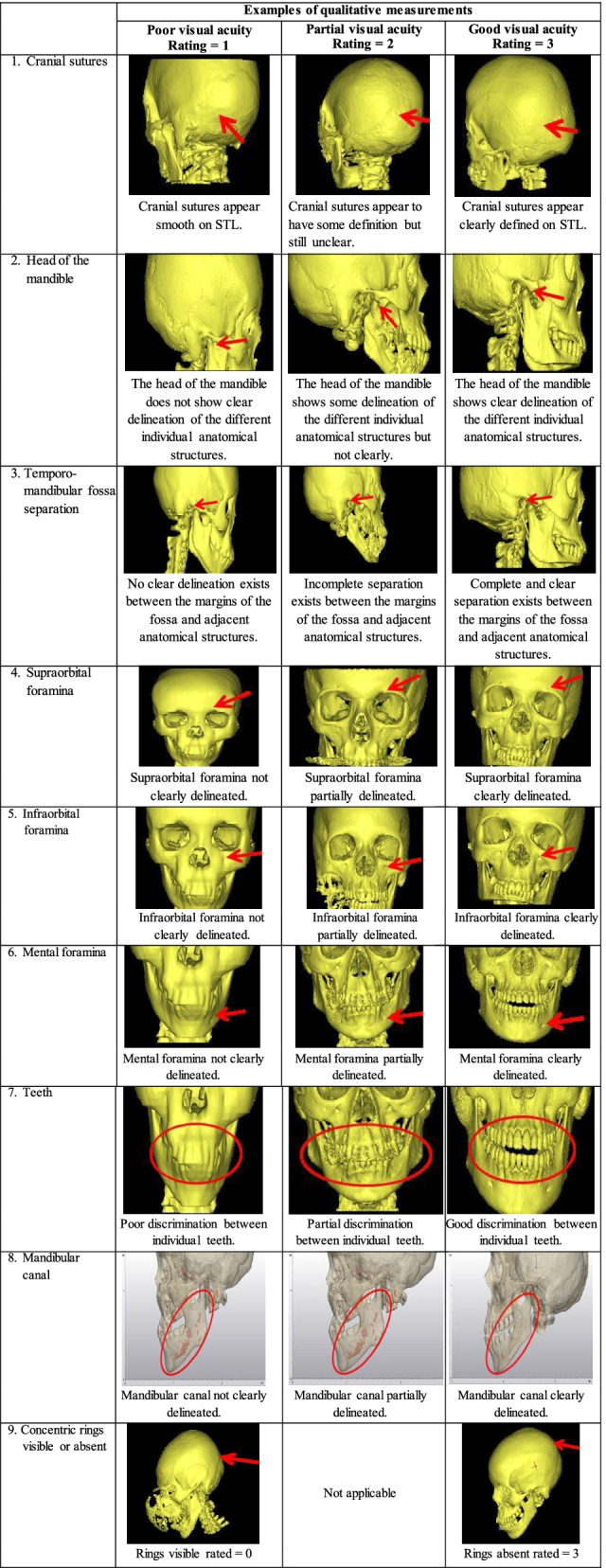
Examples of measurements of some evaluation items on the STLs

The mode and median of the scores of the eight individual CARPs of the three evaluators were grouped around the central rating of partial visual acuity. Similarly, the CARP scores were also closely grouped around the central rating of partial visual acuity. When considering the *Total CARP scores* of the three evaluators, the mean values ranged from 54.6% (13.1) to 60.0% (14.4) of the maximum possible score of 24. In contrast, the mean total *CARP + ring* scores ranged from approximately 58.5% (15.8) to 63.3% (17.1) of the maximum possible score of 27. Interestingly, ring artifacts were visible in only a few of the STLs. Furthermore, the overall impression scores of the two designers were similar. Table 5 summarises the evaluators’ measurement scores for the evaluation items of the STLs and their summary statistics.

**Table 5:** Measurement scores and summary statistics for the evaluation items of the STLs

Evaluation item	Summary statistics of STL image quality								
	Evaluator 1			Evaluator 2			Evaluator 3		
	Median	Mode	n	Median	Mode	n	Median	Mode	n
CARP 1:Cranial sutures	2	2	33	2	2	33	2	2	33
CARP 2:Head of the mandible	2	2	35	2	2	35	2	2	35
CARP 3:Temporo-mandibular fossa separation	2	2	35	2	2	35	2	2	35
CARP 4:Supraorbital foramina	2	2	29	2	2	30	2	2	30
CARP 5:Infraorbital foramina	2	2	33	2	2	33	2	2	33
CARP 6:Mental foramina	2	2	27	2	2	26	2	2	27
CARP 7:Teeth	2	2	31	1	1	31	2	2	31
CARP 8:Mandibular canal	2	2	32	2	2	32	2	2	31
Presence/Absence of rings	No: 32; Yes: 3			No: 33; Yes: 2			No: 33; Yes: 2		
CARP score	2	2	–	2	2	–	2	2	–
Mean Total CARP score	14.4	3.632	–	13.1	3.590	–	13.9	4.072	–
Mean Total CARP + ring score^a^	17.1	3.936	–	15.8	3.917	–	16.6	4.467	–
Overall impression score	–	–	–	3	3	35	3	3	35

*CARP* critical anatomical reference point

^a^Ring score refers to whether ring artifacts were present or not on the STLs. A score of 3 was allocated if present

### Evaluators’ STL image quality scoring

Four hypotheses were tested to compare the STL image quality scoring results of the different evaluators. The eight CARP Kruskal-Wallis tests performed on the three evaluators’ STL image quality scores revealed no significant differences between the three evaluators at α = 0.05 (Table 6). Similarly, for the *Total CARP score* and *Total CARP + ring score*, the differences between the scores of the three evaluators were also non-significant. When the *Overall impression scores* of the two designers were compared, the Mann-Whitney U test also revealed no significant differences at α = 0.05.

**Table 6:** Kruskal-Wallis and Mann-Whitney U test hypothesis tests for evaluator STL image quality scoring

**Hypothesis test**					
**Kruskal-Wallis**					
**Evaluation item**	**Hypothesis**	**DF**	**H statistic**	***P*-value**	**Significance**
CARP 1 score	*H1*	2	1.1809	0.55408	NS
CARP 2 score	*H1*	2	1.0042	0.60525	NS
CARP 3 score	*H1*	2	1.3301	0.51.425	NS
CARP 4 score	*H1*	2	2.9627	0.22733	NS
CARP 5 score	*H1*	2	0.7906	0.67349	NS
CARP 6 score	*H1*	2	0.8894	0.64102	NS
CARP 7 score	*H1*	2	2.3688	0.30592	NS
CARP 8 score	*H1*	2	0.1364	0.9247	NS
Total CARP score	*H2*	2	2.8907	0.93409	NS
Total CARP + ring score	*H3*	2	2.8358	0.24222	NS
**Mann-Whitney** **U** **test**					
	**Hypothesis**		**U statistic**	***P*-value**	**Significance**
Overall impression score	*H4*		554.5	0.42952	NS

*CARP* critical anatomical reference point, *DF* degrees of freedom, *NS* non-significant

### Association between overall impression and total scores

Two further hypotheses were tested to determine whether the *Overall impression score* was associated with the *Total CARP score* and *Total CARP + ring score* of the two designer evaluators. Spearman’s rank correlation calculations (*r*_*s*_) revealed that for both the evaluators, significantly strong associations were found between their *Overall impression score* and *Total CARP score*, as well as the *Total CARP + ring score* (Table 7).

**Table 7:** Spearman’s rank correlation tests for association between Overall impression score and the items Total CARP score and Total CARP + Ring score

Variable	Evaluator	Hypothesis	Spearman rank correlation	***P***-value	Strength of association	Significance
Overall impression score + Total CARP score	2	*H5*	0.6162	0.00008	Strong	S
	3	*H5*	0.6229	0.00006	Strong	S
Overall impression score + Total CARP + ring score	2	*H6*	0.6155	0.00008	Strong	S
	3	*H6*	0.6235	0.00006	Strong	S

*CARP* critical anatomical reference point, *S* significant at α < 0.05

### Classification of the STL image quality

In an attempt to categorise the different STLs according to their image quality, the classification guide was followed. According to the mean *Total CARP score*, 20% of the 35 STLs fell into the high image quality category (Table 8). However, when the STLs were categorised according to the more lenient classification of *Total CARP + ring score*, 31.4% of the STLs fell into the high image quality category. After merging the mean *Total CARP score* and the *Total CARP + ring score* STL image quality classifications, 34.3% (12 STLs) were ultimately classified into the high image quality category.

**Table 8:** Classification of STL image quality

Original STL number (n = 35)	Classification according to ***Total CARP score***					Classification according to ***Total CARP + ring score***					Final STL image quality category
	E1 Total CARP score	E2 Total CARP score	E3 Total CARP score	Mean Total CARP score over evaluators	CARP score STL classification	E1 Total CARP + ring score	E2 Total CARP + ring score	E3 Total CARP + ring score	Mean Total CARP + ring score over evaluators	CARP + ring score STL classification	
2	16	21	21	19.3	H	19	24	24	22.3	H	H
5	16	13	16	15.0	M	19	16	19	18.0	M	M
6	10	12	13	11.7	M	13	15	16	14.7	M	M
8	7	9	10	8.7	L	10	12	13	11.7	M	M
10	17	17	15	16.3	M	20	20	18	19.3	H	H
12	10	13	9	10.7	M	13	16	12	13.7	M	M
13	14	12	12	12.7	M	17	15	15	15.7	M	M
14	19	18	15	17.3	H	19	18	15	17.3	M	H
15	7	7	6	6.7	L	10	10	9	9.7	L	L
16	12	9	11	10.7	M	15	12	14	13.7	M	M
17	16	15	15	15.3	M	19	18	18	18.3	M	M
18	17	14	15	15.3	M	20	17	18	18.3	M	M
19	17	18	19	18.0	H	20	21	22	21.0	H	H
20	15	14	14	14.3	M	18	17	17	17.3	M	M
21	17	15	13	15.0	M	20	18	16	18.0	M	M
22	16	16	19	17.0	H	19	19	22	20.0	H	H
23	9	9	9	9.0	M	9	9	9	9.0	L	M
25	14	10	12	12.0	M	17	13	15	15.0	M	M
26	18	16	17	17.0	H	21	19	20	20.0	H	H
30	13	12	10	11.7	M	13	12	10	11.7	M	M
31	16	16	16	16.0	M	19	19	19	19.0	H	H
32	10	8	9	9.0	M	13	11	12	12.0	M	M
33	15	13	18	15.3	M	18	16	21	18.3	M	M
34	19	14	17	16.7	M	22	17	20	19.7	H	H
35	13	13	14	13.3	M	16	16	17	16.3	M	M
36	9	5	8	7.3	L	9	5	8	7.3	L	L
37	16	15	19	16.7	M	19	18	22	19.7	H	H
38	21	15	18	18.0	H	24	18	21	21.0	H	H
40	17	9	11	12.3	M	20	12	14	15.3	M	M
41	14	11	10	11.7	M	17	14	13	14.7	M	M
42	17	16	17	16.7	M	20	19	20	19.7	H	H
43	8	8	8	8.0	L	11	11	11	11.0	M	M
44	17	17	23	19.0	H	20	20	26	22.0	H	H
45	17	15	14	15.3	M	20	18	17	18.3	M	M
48	16	15	15	15.3	M	19	18	18	18.3	M	M

*E1, E2* and *E3* STL evaluators, *H* high STL image quality, *M* medium STL image quality, *L* low STL image quality

## Conclusion

In this study, a user-friendly SMR was developed and successfully applied to categorise 35 cranial STLs into three broad image quality categories. An extensive review of the literature confirmed that this SMR for STL image quality analysis appears to be a first of its kind. The SMR comprised several evaluation items, of which most were accompanied by a 3-point rating scale to grade the visual acuity of the STLs. After the application of the SMR, 12 of the 35 STLs were deemed to be high image quality STLs, which could be used to develop an optimal CT imaging protocol for CRPM. The metadata attached to the STLs will be used to ascertain which CT scan parameters are appropriate for such an optimised CT imaging protocol for the design and manufacturing of internal cranial prostheses. An optimised CT imaging protocol will reduce the number of resizing of prostheses, repeat CT imaging and also limit patient distress.

A user-friendly SMR was developed and used to successfully grade the image quality of STLs generated from CT scan DICOM files. The ability to grade the image quality of STLs makes it possible to plan for more accurate CT scan parameters to design and manufacture internal cranial prostheses.

## Data Availability

All data generated and analysed during this study are included in this published article.

## Abbreviations

3D: Three-dimensional
ANOVA: Analysis of variance
CARP: Critical anatomical reference point
CRPM: Centre for Rapid Prototyping and Manufacturing
CT: Computed tomography
DICOM: Digital Imaging and Communications in Medicine
HSREC: Health Sciences Research Ethics Committee
MRI: Magnetic resonance imaging
SMR: STL measurement rubric
STL: Stereolithography
